# Supplementary material for: Risk Awareness as a Key Determinant of Early Vaccine Uptake in the Mpox Vaccination Campaign in an Italian Region: A Cross-Sectional Analysis
Source: Vaccines (Basel). 2023 Nov 27;11(12):1761. doi: 10.3390/vaccines11121761 (PMC10748194; doi:10.3390/vaccines11121761)
Supplement: Supplementary file 1 [file vaccines-11-01761-s001.zip › vaccines-2697232-SI.pdf]

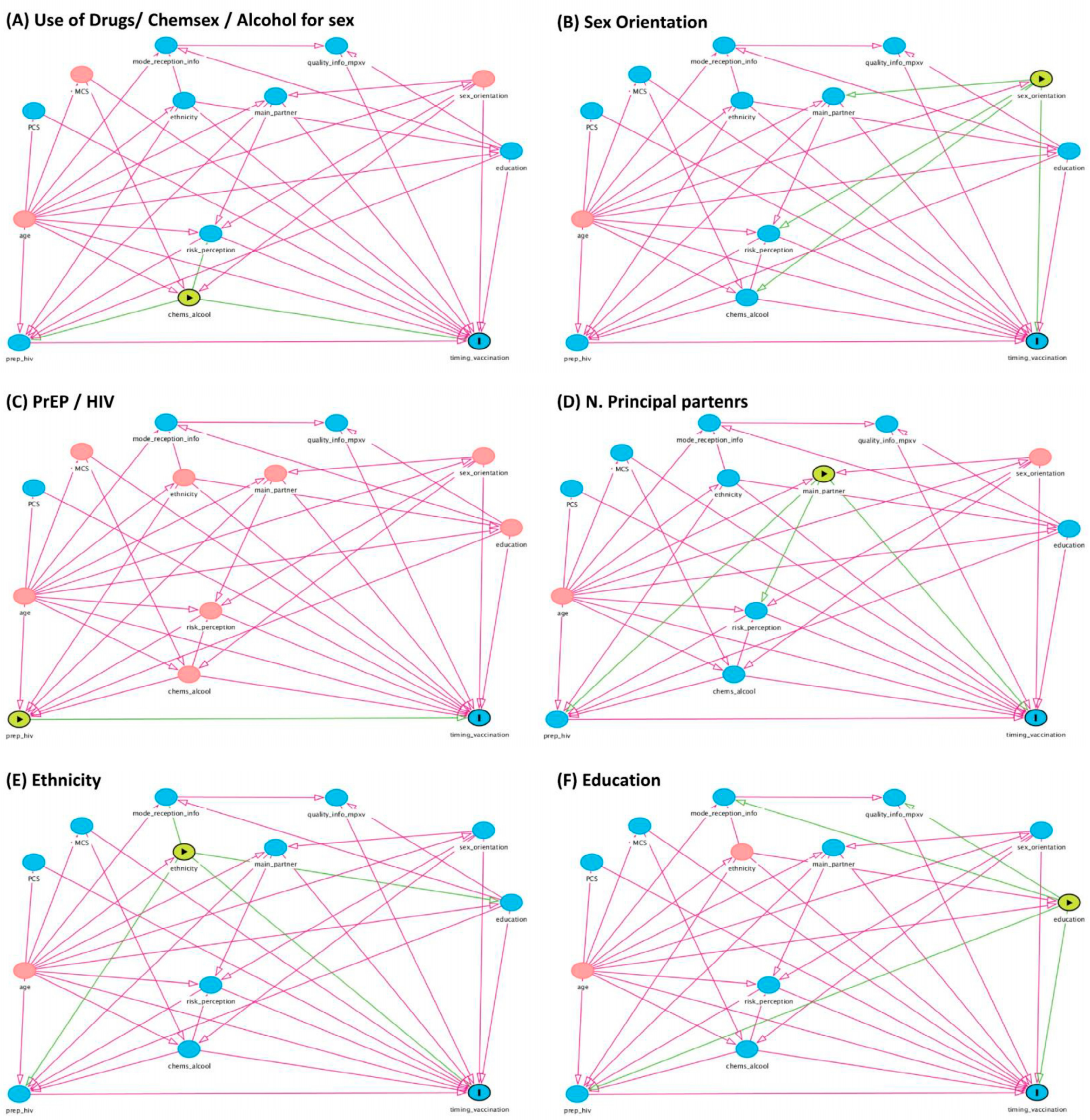
(A) Use of Drugs/ Chemsex / Alcohol for sex
(B) Sex Orientation
(C) PrEP / HIV
(D) N. Principal partenrs
(E) Ethnicity
(F) Education
mode_reception_info
quality_info_mpxv
MCS
PCS
ethnicity
main_partner
sex_orientation
education
age
risk_perception
chems_alcool
prep_hiv
timing_vaccination

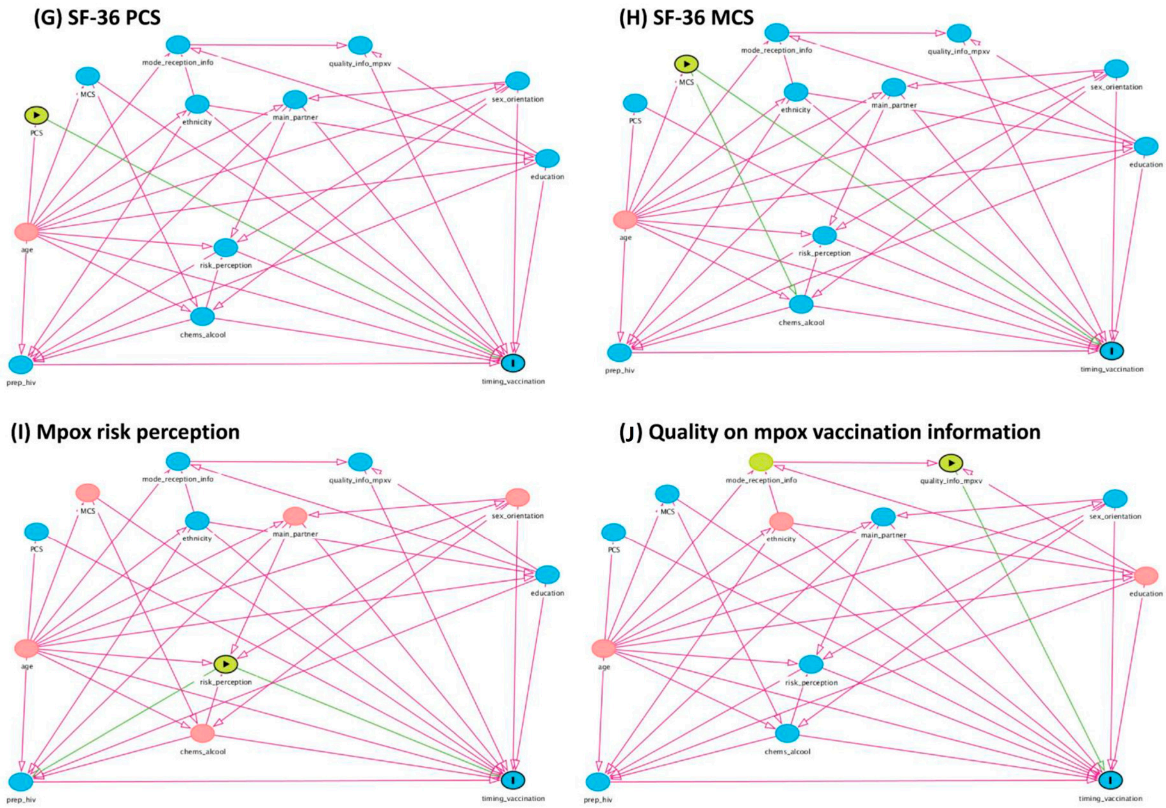

Supplemental Figure S1. Directed acyclic graphs (DAGs) depicting the assumed underlying causal paths between the different exposures of interest (**A**–**J**) and the two binary outcomes of vaccination timing. Age is unconfounded by definition, but in order to increase the precision of the estimates, logistic models have been adjusted for a strong adjusted for a strong predictor of the outcome as age.
